# Radiation Source Localization Using a Model-Based Approach

**DOI:** 10.3390/s23135983

**Published:** 2023-06-27

**Authors:** András Molnár, Daniel Kiss, Zsolt Domozi

**Affiliations:** 1 Faculty of Economy, J. Selye University, Bratislavská cesta 3322, 945 01 Komárno, Slovakia; 2 John von Neumann Faculty of Informatics, Obuda University, Becsi ut 96/b, 1034 Budapest, Hungary; kiss.daniel@nik.uni-obuda.hu; 3 Doctoral School of Applied Informatics and Applied Mathematics, Obuda University, Becsi ut 96/b, 1034 Budapest, Hungary; domozi.zsolt@phd.uni-obuda.hu

**Keywords:** gamma radiation, drone, field dose distribution

## Abstract

The procedure is practically an optimization method, during which it is assumed that the gamma dose values detected at different points above the area come from the background radiation and from a single source found in the area. Accordingly, the procedure searches within the area for a geographical coordinate for which the distance law for the spatial propagation of radiation will be true. In order to validate the procedure, we performed measurements in a test area in such a way that all parameters of the source, including its location, were well defined. However, these data were not taken into account during the processing, i.e., the search procedure did not have these data. We can estimate the radiation position without a positional parameter. The exact coordinate and the intensity of the radiating sample were only used when checking the results. We have also applied the method to the raw data of our experiments carried out in the past if we used one source for them. The results confirmed our assumptions. The method is suitable for determining the starting parameters of more complex processes that can even detect multiple sources, but assuming one source, it has proven to be a reliable analytical method on its own.

## 1. Introduction

The localization of open-air gamma radiation sources can be performed plainly using imaging devices sensitive in the gamma range. Such imaging devices include, among others, gamma cameras used in the medical field or their various upgraded versions [1,2]. With these devices, a prerequisite for imaging is the use of an optical imaging system operating in the gamma range, such as the pinhole “optics” fitted to a gamma camera or the so-called collimator [3,4]. Both are made of lead and are extremely heavy. Given that the project aims to carry out the localization of the source using a cheap, portable, and quickly deployable drone platform-mounted detector, the detector must be lightweight. Consequently, conventional imaging tools sensitive to the gamma range are not applicable to this task.

Provided that the characteristics of the pixels generated during imaging are defined by the intensity of the pixel (on occasion by intensity broken down into color channels) and by the position of the pixel (the X; Y coordinates interpreted on the even image), then we can conclude that the intensity is provided by the signal from the radiation-sensitive sensor whereas the position of the pixel is provided by the imaging optics. Consequently, the imaging system can be divided into two basic units, one for measuring the intensity of the measured radiation and one for determining the measurement coordinates [5].

In light of the above, there is significant potential for imaging without an optical imaging system in the classical sense. In the project, the intensity of the measured radiation was realized using a Geiger-Müller counter, and during other experiments, with scintillation detectors. The measurement coordinates were determined using a GPS positioning device. The complex system assigns to each measured coordinate value the intensity of the currently measured radiation, i.e., the number of gamma photons detected by the detector [6]. During the course of the experiments, we initially used a GPS device with a measuring frequency of 1 Hz and later one that of 5 Hz. Accordingly, the number of gamma photons was matched to the measurement period of the GPS and the measured values were converted from that to the number of events per second (Count per Second CPS).

Based on the data of the proposed detector system, it is not possible to create an image directly in the gamma range. Additional processing is required to create the image. During gamma-range tests, the intensity of the source to be detected is very low, barely distinguishable from the ubiquitous background radiation. In other words, this means a very poor signal-to-noise ratio. During post-processing, a priority task is to highlight the information from the measurement data loaded with such high noise.

We developed a successful solution to produce the color-coded dose distribution map of the examined area using integration divided into units of area and interpolation between units of area [7,8,9,10,11]. At the same time, the method is very sensitive to the size of the cell units determined during processing [12]. In order to visualize the measured values as accurately as possible, a new procedure was needed which is effective, but fundamentally different from the applied method. One such possible control method is the correlation-based processing of the measurement data. Since this method does not apply the unit division of areas, its result can be used for the automated determination of the size of elementary areas during the area division. However, in the case of the localization of a source, the correlation method in itself is also applicable [13,14].

## 2. Materials and Methods

### 2.1. Mathematical Model of Spatial Intensity Distribution

Radiation source localization based on physical models and simulation is an actively researched area [15,16]. To formulate a mathematical model capturing the intensity distribution measured in the vicinity of a single-point radiation source [17], we made the following assumptions. First, we assumed that measurements cover only a relatively small area, therefore the curvature of the Earth can be ignored in the calculations. This lets us represent our data M as a set of observations, each measurement having the form $m_{k}=\left(x_{k},\;y_{k},\;i_{k}\right)$ where $x_{k}$ and $y_{k}$ give the two-dimensional spatial coordinates of the observation, and $i_{k}$ is the intensity measured by the probe above a given location. The description allows multiple intensity measurements for the same spatial location, which might occur as a result of the actual flight path. We also assumed that the source is a single point $s=\left(s_{x},\;s_{y}\right)$, not necessarily inside the actual measurement zone, and the radiation is isotropic. Our third assumption was that the altitude of the device is negligible during the flight compared to the spatial dimensions of the measurement zone, therefore the distance between the probe and the radiation source is vanishing once the device hovers precisely above s.

**Figure 1:**
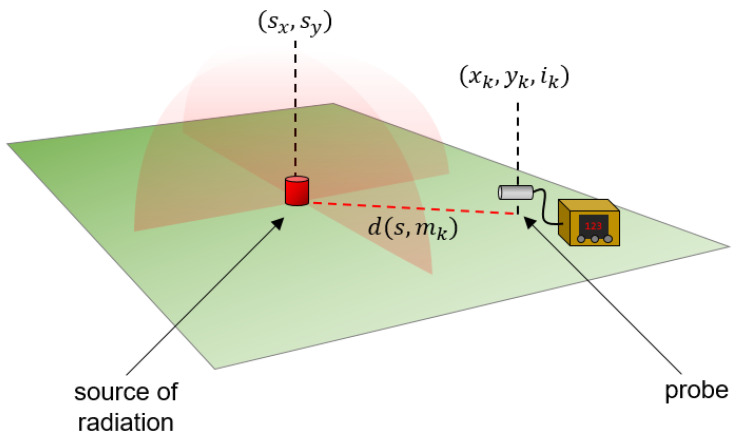
Schematic figure of the idea behind the mathematical model. The intensity measured by the probe is inversely proportional to the distance from the unknown source point of radiation, thus, the coordinates of the source point can be found by minimizing the difference between the observed and modeled intensity measurements.

As shown on Figure 1, as an electromagnetic wave, gamma radiation emitted by an isotropic radiator follows an inverse-square law, therefore the intensities, expressed in counts per unit time, in different locations measured over the flight should reflect this law. Given a true (hypothetical) location of the radiation source $\left(s_{x},\;s_{y}\right)$, we model the measured intensities as

(1) $${\hat{i}}_{k}=\frac{\mu_{p}}{d\left(s,\;m_{k}\right)}+\varepsilon_{k},$$

where $d\left(s,\;m_{k}\right)$ is the distance between the position of the radiation source s, and the position of measurement $m_{k}$, while $\mu_{p}$ is a proportional factor that scales radiation intensity to counts per unit of time recorded by the probe, and $\varepsilon_{k}$ is a term to describe random noise (e.g., the contribution of natural background radiation). Using our first assumption above, $d\left(s,\;m_{k}\right)$ is considered here as the Euclidean distance of geographical coordinates.

Now, given a set of actual measurements M and an unknown source of radiation $\hat{s}$, we compute the prediction error of the model as

(2) $$e\left(M,\hat{s}\right)=\overset{\left|M\right|}{\underset{k=1}{\sum }}{\left({\hat{i}}_{k}-i_{k}\right)}^{2}.$$

Using the previous formula for ${\hat{i}}_{k}$, we can rewrite the equation as

(3) $$e\left(M,\;\hat{s}\right)=\overset{\left|M\right|}{\underset{k=1}{\sum }}{\left(\frac{\mu_{p}}{\sqrt{{\left({\hat{s}}_{x}-x_{k}\right)}^{2}+{\left({\hat{s}}_{y}-y_{k}\right)}^{2}}}-i_{k}+\varepsilon_{k}\right)}^{2}$$

which can be minimized by finding optimal choices for ${\hat{s}}_{x},\;{\hat{s}}_{y}$ and $\mu_{p}$, corresponding to the location and the intensity of the radiation source (Figure 1).

In theory, and especially in the absence of the $\varepsilon_{k}$, the problem is trivial, although, the significant contribution of noise makes the minimization more challenging in practice, as many standard optimization procedures will likely stop in local optima. To avoid this situation, a stochastic approach, more precisely, an evolutionary algorithm implemented in Excel’s Solver add-on [18] was used to find the best approximation for the values of ${\hat{s}}_{x},\;{\hat{s}}_{y}$ and $\mu_{p}$. The lower and upper bounds for the coordinates were set as ${\hat{s}}_{x}^{L}=\underset{k}{\min}\;\left\{x_{k}\right\}$, ${\hat{s}}_{x}^{U}=\underset{k}{\max}\;\left\{x_{k}\right\}$, and ${\hat{s}}_{y}^{L}=\underset{k}{\min}\;\left\{y_{k}\right\}$, ${\hat{s}}_{y}^{U}=\underset{k}{\max}\;\left\{y_{k}\right\}$, respectively. The lower bound of $\mu_{p}$ was set to 0, but as no information was available for the possible upper limit, preliminary executions were performed to obtain a realistic value for $\mu_{p}^{U}$. We tested the optimization method with multiple parameter settings (i.e., population sizes and mutation rates) and found an ideal population size to be 100 and a mutation rate to be 0.2 for our data sets with approximately 2000 observations.

### 2.2. Instruments Used for the Experiments

In the experiments, two radiation monitoring detectors were used. One of the detectors was built on the basis of a Geiger-Müller (GM) counter [19]. This detector system essentially detects only ionizing radiation, in this case, the gamma particle (radiation). The events detected during a unit of time can be considered proportional to the radiation dose. The detector is not capable of determining the energy of the gamma particle. The advantage of the GM detector system is that its signals can be easily processed, in this way, it is sufficient to use a microcontroller with small computing power on board. Its disadvantage is its relatively high supply voltage requirement (GM tubes require a supply voltage of 700–900 V, depending on the type).

The other detection method is based on the scintillation detector [20]. The energy of the detected gamma particle can also be measured with this detector. The additional information helps in the selective detection of a specific sample. Another advantage of the scintillation detector is its high sensitivity despite its relatively small size. Nonetheless, the disadvantage of the detector is that a large amount of data is generated from energy measurement, as well as the post-processing of native data. On account of the determination of the energy level, a microcontroller with slightly higher computing power is needed compared to GM detector systems.

**Figure 2:**
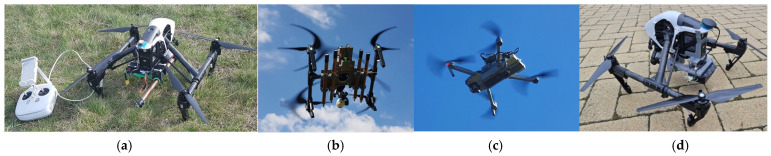
Drone-fitted experimental devices for dose distribution measurements in the field (pictures order is: (**a**–**d**) Inspire I with one GM-tube detector (**a**), Inspire I with four GM-tube detectors (**b**), Mavis Pro with small scintillation detector (**c**), Inspire I with scintillation detector suitable for fast spectrum analysis (**d**).

As for the carrier device, due to the large GM tubes, a multicopter was chosen that could fit the detector system on board. DJI’s Inspire I multicopter (Figure 2a,b) is reliable and has a sufficient load capacity. It is capable of automatic route flight, which makes it a convenient choice to scan the experimental area.

The Mavic is also a DJI product (Figure 2c), which was fitted with a small scintillation detector. Similar to Inspire I, the Mavic is also capable of pre-programmed route flight, but due to its smaller size, it can be transported and handled more conveniently. Unfortunately, the carrying capacity of the Mavic is severely limited, in this way, it cannot transport a larger scintillation detector for a minimum of 10 to 15 min in terms of experiments. The sensitivity of the installed detector equals that of the detector system with four GM tubes (Figure 2b), in this way, it is a suitable alternative to the large 4-tube Inspire I carrier. Although the scintillation detector is also suitable for determining the energy of the detected gamma particle, The device shown in Figure 2c works in particle counter mode so that there is no need to use an on-board micro-controller with high performance and thus with higher energy demand.

In order to fully exploit the potential inherent in scintillation detectors, a special detector was developed [7,8,9,10]. The sensitivity of this detector enables it to provide a statistically processable amount of measurement data per second, also including energy levels. However, the mass of the detector is already too large for the DJI Mavic, so it was once again placed onboard the already proven DJI Inspire I (Figure 2d).

## 3. Results

### 3.1. The General Characteristics of the Measurements

The measurements were carried out using autonomous flight mode, with detectors mounted on various drones [21,22]. The height of the flights above the samples was 5–6 m. The reference points were recorded with the measuring device, as the final stage of the experiment, without turning off the device. This method ensured that the coordinates were loaded with identical errors during the measurement, in this way their relative accuracy was as high as possible. The average static position error of the GPS device is 3 m, but during the course of the experiment, the accuracy of the coordinates in relation to one another was 0.2 m. During the evaluation of the measurement data, the measurement data of the reference point were removed. During the evaluation of the determined source, only the detection values of the gamma particles collected during the active measurement and the coordinates assigned to them were taken into account. The reference points shown in the figures are indicated to visually depict the accuracy of the measurement. The test areas were 30 × 90 m in size with slight variations.

### 3.2. Carrying Out Measurements

**Figure 3:**
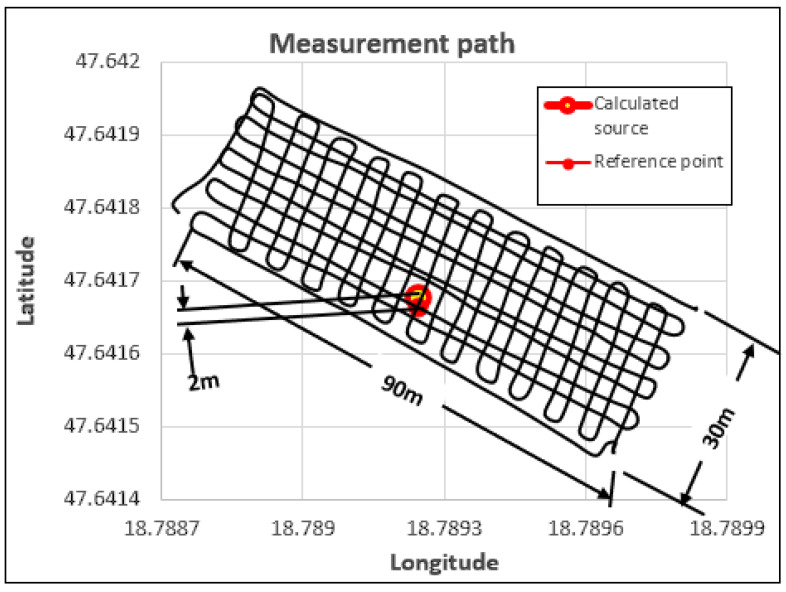
Evaluation of the native data of the GM tube measurement performed in the test area with continuous scanning flight mode.

GM tube detectors exclusively allow the detection of gamma particles. GM tubes do not provide information on the energy of the particle. Provided that the searched source causes a significant increase in detection distance compared to the background radiation, the sample can be correctly localized in the investigated area by using the coordinates linked to the detections [7,8,9,10,11]. During the experiment, four LND 7808-type GMs [23] were installed on the drone. During the measurement, the sample on occasion increased the detection value of the detector to a value of 30 detections per second—slightly more than double compared to the average 13 detection values of background radiation per second. Since this increase in the number of detections alone is sufficient to detect and localize the source, it is very much suitable for testing processing procedures. Figure 3 illustrates the detected source and the real position of the source marked with the reference point based on the processed measurement data gained during the scan flight of a drone equipped with such a detector system. It is clearly visible that the measured and actual positions are very close to one another. This accuracy is perfectly sufficient for a ground team to physically locate the discovered radiation source and begin the removal work. Raw data recorded during the flight were used for the evaluation. From the data, only the parts recorded before take-off and in the time after landing were removed, which are irrelevant and at the same time disturbing from the point of view of the scanning measurement. No filtering, noise removal, or other pre-processing was performed on the recorded data [24].

**Figure 4:**
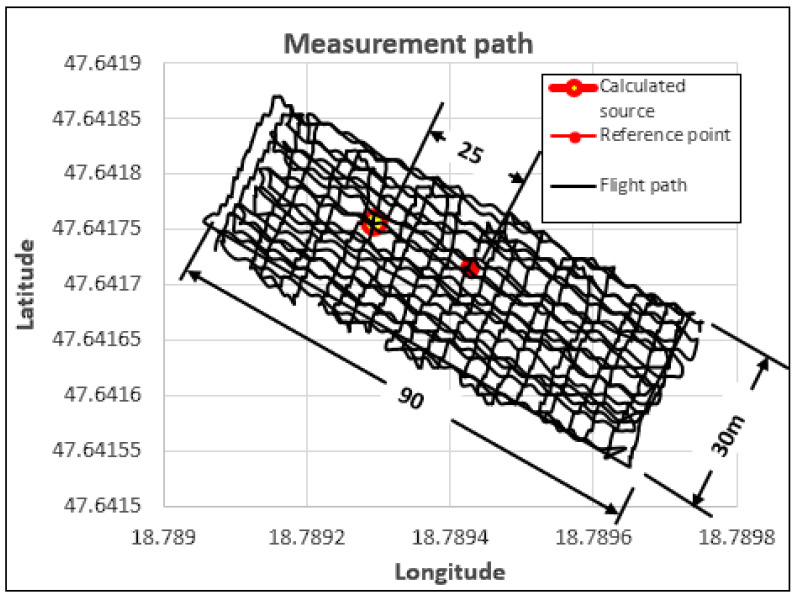
Evaluation of the native data of the scintillation detector measurement performed in the test area with continuous scanning flight mode.

In Figure 4, the scanning measurement was carried out using a drone equipped with a scintillation detector [25,26,27]. The purpose of the experiment was to be able to detect the low-activity sample placed in the area during a continuous scanning flight. The scintillation crystal was a 21.5 × 50 × 50 mm CsI (Tl) (Thallium Activated Cesium Iodide). There was no significant difference visible in the native measurement data during the measurement. The excess radiation caused by the sample merged into the detections caused by the background radiation. In contrast to the GM tube detectors, the scintillation detector made it possible to determine not only the interference of the gamma photon but also its energy level [28,29]. Figure 4 shows the result of processing the native data measured during the experiment. During the processing, we did not apply the energy-based classification of the detections. The detections of the scintillation detector were treated similarly to the GM tube detector, that is to say, the number of particles detected per unit of time was taken into account (Count per Second (CPS)). Despite the weakly detectable sample, the processing procedure was able to detect a certain degree of clustering in the measurement data in the test area, i.e., a positive deviation compared to the radiation values measured in the area. It can also be read from the figure that there is a significant difference in distance between the calculated and the real position (reference point) of the sample.

**Figure 5:**
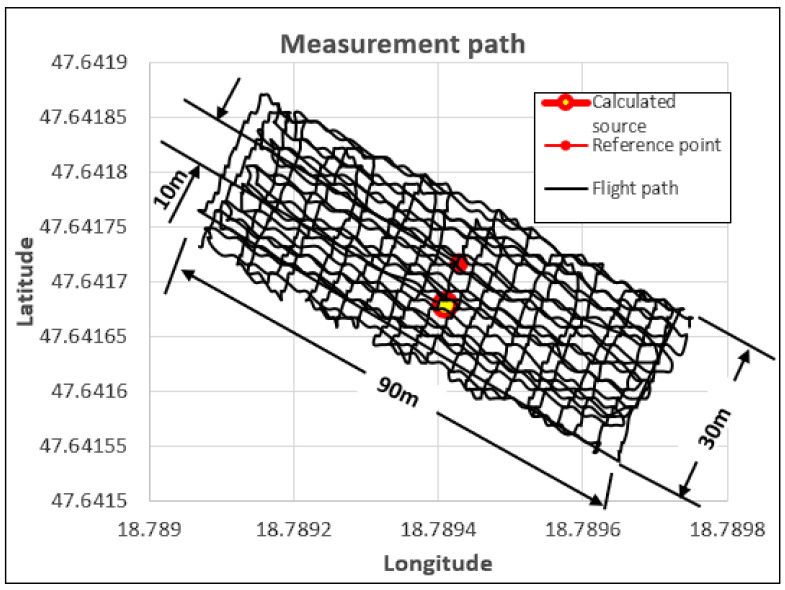
Evaluation of the data of the scintillation detector measurement performed in the test area, with continuous scanning flight mode, cleaned of divergent noise.

One reason for the position deviation visible in Figure 4 is the amount of noise generated during the measurement, the other is the low detection increase resulting from the low activity of the tested sample. By performing a time series analysis on the native measurement data, it was established that unrealistically high detection values were recorded on several occasions. These values only appeared for a very short time and based on their value; they can be excluded from the measurement data with great certainty. Figure 5 illustrates the processed result of the unfiltered data presented in Figure 4, after the automatic deletion of unrealistically high (outlier) detection values. The figure clearly shows that the position of the calculated radiation source is significantly closer to the real (reference) position. Given that disturbing noises can be marked with simple algorithmic procedures, their removal can be automated. For the noisy data series, the procedure was not implemented by replacing the average of the surrounding data, but by their complete erasure. In the course of the experiments, it was clearly established that filtering out noise with large deviations is necessary in terms of the localization of the source. During the experiments, the position of the localization clearly became more accurate using the denoised data.

**Figure 6:**
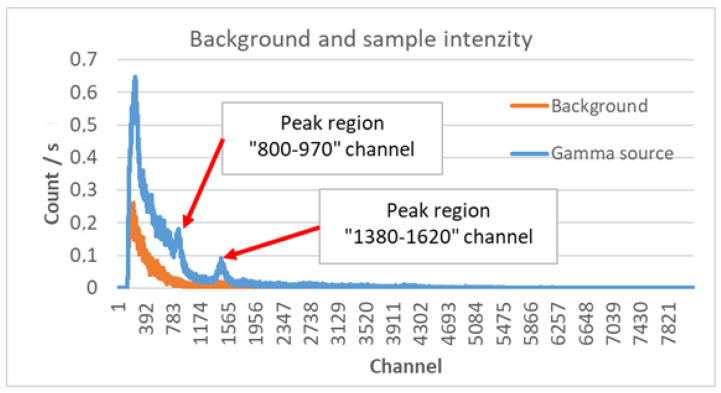
Static measurement to determine the special spectrum of the tested sample.

The position error is caused by the low number of detections in connection with the low-activity samples of the other source already mentioned. This in fact means no or a barely noticeable increase in the average number of detections of background radiation in the native data. In other words, the signal-to-noise ratio is not high enough, and the extraction of useful information presents difficulties. When a scintillation detector is used, it is also possible to determine the energy of the detected gamma photon. It is known that the energy of gamma photons produced during the decay of a given isotope shows an energy spectrum characteristic of the isotope. If we are looking for a radiation source of known material quality, the radiation spectrum characteristic of the isotope to be detected can be used [30,31]. In Figure 6, the spectrum of the Autunite mineral used during the experiments was mounted on the drone together with the detector [32]. Uncalibrated detector data can be seen on the diagram. The diagram shows the number of detections per second matched to the channels belonging to the background radiation (Figure 6, orange diagram) and to the sample (Figure 6, blue diagram). Since the detector was not calibrated, only the channel numbers are displayed on the diagram instead of the energy values [33]. Given that the measurements are comparative, and their interpretation is only qualitative, the lack of calibration does not cause interpretation problems. Figure 6 shows two typical peaks of the tested sample. We also marked their surroundings. One peak is located between channel numbers 800 and 970 and the other between channel numbers 1380 and 1620. Even in the case of low intensity, it can be assumed that the number of detections caused by the sample in the indicated channel ranges will be detectable in a higher proportion compared to the other detectable channel ranges.

**Figure 7:**
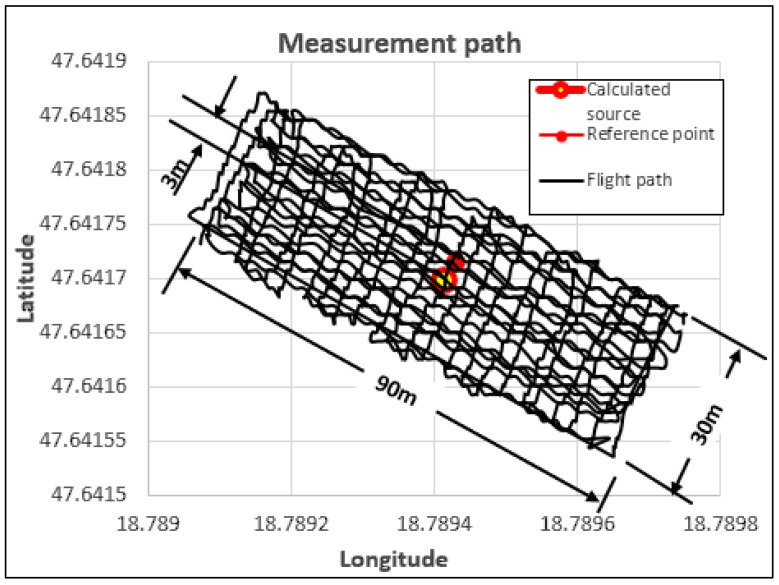
Evaluation of the energy-selective native data of the scintillation detector measurement performed in the test area with continuous scanning flight.

Figure 7 illustrates the calculated and actual position of the radiation source determined by processing the data of the channel windows equivalent to the previously discussed energy windows from the data presented in Figure 4. In this case, the number of detections per given time unit decreased significantly, but due to the already-mentioned improvement in the signal-to-noise ratio, we reached a more favorable evaluation result. It can be seen in the figure that the location of the calculated radiation source closely approximated the real position, in this way it meets the expectation that based on the coordinates obtained in this way, a ground team can find the source with great certainty.

## 4. Discussion

The presented localization procedure was examined with the data from several old and new surveys. GM tube and scintillation detector measurements were also reworked. In the case of all measurements where the signals of the sought samples clearly stand out from the signals produced by the background radiation, the procedure reliably determined the geographic coordinates of the sought sample in accordance with the localization accuracy of the GPS system used. The accuracy was practically within 3 m. We achieved the same localization accuracy in the case of weak sources that could not be detected from the examined distance with traditional measurements, but knowing the energy spectrum characteristic of the sample, we applied so-called energy-selective pre-processing. The accuracy of the procedure is not affected by the geometry of the flight path used during the measurement.

The developed method was compared with our previously used, self-developed area-based distribution model [7,8,9,10,11]. In the case of our cited model, the result of the processing depends to a large extent on the definition of the appropriate area unit. Since we could not provide an exact mathematical method for the optimal area division, we relied on our empirical results. Accordingly, we set the size of the processing elementary area unit to the measurement distances determined during the flights. In the case of the method presented in this thesis, there is no need to divide the area. The localization accuracy of the procedure coincides with the accuracy of the GPS device used during the flight. Therefore, in the case of a source, the determination of the position of the source can be considered as a reference value. Using this reference value, we set the parameter of our method requiring area division (determining the size of the area unit to be used during the calculations) in such a way that it gives the localization of the source given by this method. After that, by processing the data of additional unknown areas taken with the same flight conditions while maintaining the area parameter, we can consider the localizations of the sources as real even in the case of multiple sources in the case of our old model.

## Data Availability

Data sharing not applicable.
